# Phytochemical Analysis, Antioxidant Activity, Fatty Acids Composition, and Functional Group Analysis of *Heliotropium bacciferum*

**DOI:** 10.1155/2014/829076

**Published:** 2014-11-12

**Authors:** Sohail Ahmad, Shabir Ahmad, Ahtaram Bibi, Muhammad Saqib Ishaq, Muhammad Siddique Afridi, Farina Kanwal, Muhammad Zakir, Farid Fatima

**Affiliations:** ^1^Department of Chemistry, Kohat University of Science and Technology, Kohat 26000, Pakistan; ^2^Department of Chemistry, Islamia College University, Peshawar 25120, Pakistan; ^3^Department of Microbiology, Abasyn University, Peshawar 25000, Pakistan; ^4^Medicinal Botanic Centre, PCSIR Labs Complex, Peshawar 25120, Pakistan

## Abstract

*Heliotropium bacciferum* is paramount in medicinal perspective and belongs to Boraginaceae family. The crude and numerous fractions of leaves, stem, and roots of the plant were investigated for phytochemical analysis and DPPH radical scavenging activity. Phytochemical analysis of crude and fractions of the plant revealed the presence of alkaloids, saponins, tannins, steroids, terpenoids, flavonoids, glycosides, and phenols. The antioxidant (free radical scavenging) activity of various extracts of the* Heliotropium bacciferum* was resolute against 2,2-diphenyl-1-picrylhydrazyl (DPPH) radical with the avail of UV spectrophotometer at 517 nm. The stock solution (1000 mg/mL) and then several dilutions (50, 100, 150, 200, and 250 mg/mL) of the crude and fractions were prepared. Ascorbic acid was used as a standard. The plant leaves (52.59 ± 0.84 to 90.74 ± 1.00), stem (50.19 ± 0.92 to 89.42 ± 1.10), and roots extracts (49.19 ± 0.52 to 90.01 ± 1.02) divulged magnificent antioxidant activities. For the ascertainment of the fatty acid constituents a gas chromatograph hyphenated to mass spectrometer was used. The essential fatty acids for growth maintenance such as linoleic acid (65.70%), eicosadienoic acid (15.12%), oleic acid (8.72%), and palmitic acid (8.14%) were found in high percentage. The infrared spectra of all extracts of the plant were recorded by IR Prestige-21 FTIR model.

## 1. Introduction

Medicinal plants and their therapeutic values are extensively used for an array of diseases all over the world. Divergent chemical constituents isolated and characterized from plant species of Boraginaceae family include flavonoids, pyrrolizidine alkaloids, naphthoquinones, phenols, and terpenoids. From different parts of various plants significant pharmacological and biological activities have been reported previously. The biological activities of constituents revealed antitumor, anti-inflammatory, antiviral, antiplatelet, cardiotonic, wound healing, contraceptive, prostaglandin, and wound healing properties [1]. Among foremost health problems, infectious diseases account for 41% of the global disease burden along with noninfectious diseases (43%) and injuries (16%) [2]. A rich source of pyrrolizidine alkaloids is present in* Heliotropium bacciferum *of family Boraginaceae, some of which have antihyperlipidemic, antitumor, antidiabetic, and antimicrobial properties [3]. Due to the biological activities of the plant antioxidants against reactive oxygen species, such as hydrogen peroxide and superoxide, they have profound significance. Reactive oxygen species (ROS) induce oxidative damage to biomolecules such as carbohydrates, lipids, proteins, and nucleic acids. The oxidative damage causes many diseases such as arteriosclerosis, rheumatoid arthritis, ageing, cancer, and cirrhosis [4]. Because of radiations, chemicals, environmental pollutants, toxins, spicy and deep fried food, and physical stress, free radicals cause change in gene expression, depletion of immune system antioxidants, and abnormal proteins induction. For the production of free radicals in food, living systems, and drugs, oxidation process is one of the most significant routes. Hydroperoxidase and catalase enzymes convert hydroperoxides and hydrogen peroxides to nonradicals and in human body act as natural antioxidants [5]. Several biological mechanisms of polyphenolic substances have been credited to the metal chelating properties or reducing properties of antioxidants [6, 7]. In food nutrition assessment, fatty acids have gained significance in the diagnosis of various diseases and pharmacology [8–10] due to biological importance [11, 12]. In lowering risks of inflammation, heart diseases and, for immunity enhancement, saturated fatty acids either monosaturated or polysaturated have been used [13–18]. For fatty acids determination different analytical techniques have been used which contain spectrophotometric, HPLC [19–21], enzymatic, and gas chromatography (GC) [22, 23]. For the analysis of fatty acids, GC-MS, due to different reasons such as resolution, sensitivity, and speed, was the scheme of choice [24, 25]. The present study was therefore designed to investigate the phytochemical and GC-MS analysis, antioxidant activities, and FTIR spectra of methanol, *n*-hexane, ethyl acetate, *n*-butanol, and aqueous extracts of the plant* Heliotropium bacciferum*.

## 2. Materials and Methods

### 2.1. Plant Collection and Identification

*Heliotropium bacciferum* was collected from district Karak, Khyber Pakhtunkhwa, Pakistan, and then was identified by plant taxonomist in the Department of Plant Sciences, Kohat University of Science and Technology (KUST), Pakistan.

### 2.2. Extraction and Fractionation

The plant leaves, stem, and roots were shade-dried, crushed, and milled into powder form. The coarse power (500 g) of each part was taken and macerated in methanol for 15 days by the same method as that of Allen Jr. et al. [26]. After maceration, the soluble methanol fraction was filtered and concentrated under vacuum as a consequence of Rotary vacuum evaporator (PLC/MBC (Phy. Std.)/011 Eyela) at 40°C. The crude methanol extract (80 gm) of each part was then suspended in distilled water (500 mL) and partitioned in succession with *n*-hexane, ethyl acetate, *n*-butanol, and water.

### 2.3. Ash Value

The method of Premnath et al. [27] was employed for the determination of ash value of the plant* Heliotropium bacciferum*. Furnace PLC/MBC/W1/32 was used for the determination of ash value.

### 2.4. Moisture Value

For the determination of moisture value of the plant, the method of Ashutosh et al. [28] was used. For moisture value determination, Oven PLC/MBC/W1/21 was used.

### 2.5. Extractive Value

The extractive values of all the five (5) extracts of the leaves, stem, and roots of plant* Heliotropium bacciferum* were determined by the method of Singh et al. [29].

### 2.6. Preliminary Phytochemical Screening

Qualitative tests were performed on different extracts of leaves, stem, and roots of the plant by employing standard protocols [30–32] for the detection of carbohydrates, saponins, alkaloids, tannins, terpenoids, steroids, flavonoids, and so forth.

### 2.7. Diphenyl Picryl Hydrazine (DPPH) Radical Scavenging Activity (Antioxidant Activity)

The DPPH radical scavenging activity of the crude and various fractions of leaves, stem, and roots of* Heliotropium bacciferum* were determined by UV spectrophotometer at 517 nm in opposition to 2,2-diphenyl-1-picrylhydrazyl (DPPH) radical. The antioxidant activity was resolved by the procedures described in the past [33] with slight modifications. Stock solution (1000 mg/mL) of extracts of* Heliotropium bacciferum* was prepared; then dilutions of the crude and fractions (50, 100, 150, 200, and 250 mg/mL) were prepared. As a standard, vitamin C (ascorbic acid) was used. For comparison, dilutions (50, 100, 150, 200, and 250 mg/mL) of ascorbic acid were also prepared. Solution of DPPH (0.003 g/100 mL) was prepared and then this solution was added to each of the five dilutions of the plant extracts. The absorbance was calculated after 30 minutes at 517 nm by spectrophotometer. The increase in the DPPH free radical scavenging activity is attributed to the decline in the absorbance of the DPPH solution. Then the percent radical scavenging activity (% RSA) was calculated by the following formula:

(1) % RSA =Absorbance  of  DPPH−Absorbance  of  SampleAbsorbance  of  DPPH.

### 2.8. Fatty Acids Quantification of* Heliotropium bacciferum* by Gas Chromatography Mass Spectrometry (GC-MS)

#### 2.8.1. Chemicals and Reagents Used

Methanol (10%), boron trifluoride solution (BF_3_), 0.5 N methanolic sodium hydroxide (NaOH) solution, *n*-hexane, sodium chloride (NaCl), fatty acid methyl esters (FAMEs), helium gas (99.99%), tridecanoic acid methyl ester, and *n*-hexane extract of the plant were used.

#### 2.8.2. Preparation of Standards

For the preparation of internal standard, in 1 mL hexane, 13.7 mg tridecanoic acid methyl ester was dissolved. 10 mg of *n*-hexane extract was diluted in FAMEs mix standard (10 mL) with dichloromethane (CHCl_2_) for preparation of external standard.

#### 2.8.3. Methodology Used in GC-MS Technique

A gas chromatograph (Shimadzu) hyphenated to mass spectrometer QP 2010 plus (Tokyo, Japan) outfitted with an autoinjector (AOC-20i) and autosampler (AOC-20S) was used. As a carrier gas, helium was used. On a capillary column (TRB-FFAP; Technokroma) having specifications, i.d., 0.35 mm, length, 30 m, thickness, 0.250 *μ*m, all chromatographic separations were performed. Fatty acids (FA) are polar compounds and are not volatile. The sample analyzed must be volatile for gas chromatographic technique. GC-MS procedure was used for fatty acids investigation. Methylation is focal procedure used for the conversion of nonvolatile fatty acids (FA) into volatile fatty acids methyl esters or FAMEs [34].

The standard procedure was used for determination of fatty acid contents [35]. In 25 mg sample, 0.1 mL internal standard and 1.5 mL methanolic NaOH (0.5 N) were added. The solution was heated for 5 minutes on hot plate in boiling water. The sample was then cooled and 10% CH_3_OH and 2.5 mL BF_3_ solution were added. Sample solution again was potted and in boiling water on hot plate heated for about 30 minutes. Then cooled and saturated NaCl solution (4 mL) was added to the esterified solution and extracted twice with hexane (1 mL), filtered by 0.45 micrometer (*μ*m) membrane filter and subjected to GC-MS scheme.

### 2.9. FTIR (Fourier Transform Infrared Spectroscopy) Study of Plant Extracts

IR Prestige-21 (Shimadzu Japan) FTIR model was used with IR Solutions software [36]. The scheme used by Meenambal et al. [37] was carried out for all the plant extracts in dried form by FTIR spectroscopy.

## 3. Results

### 3.1. Moisture, Ash, and Extractive Values

The moisture value of the whole plant was 12% and the ash value was 8.67%. The plant extractive values were calculated separately for all the five (5) extracts of leaves, stem, and roots. Methanol extract of leaves, stem, and roots had high percentage of extractive values shown in Table 1.

### 3.2. Phytochemical Screening

Phytochemical screening of various extracts of the leaves, stem, and roots of plant* Heliotropium bacciferum* revealed the presence of steroids, tannins, alkaloids, saponins, glycosides, terpenoids, phenols, and flavonoids (Table 2). In all plant extracts alkaloids were present. Except *n*-hexane fraction, saponins were present in all plant extracts.

### 3.3. Diphenyl Picryl Hydrazine (DPPH) Radical Scavenging Activity (Antioxidant Activity)

**Figure 1:**
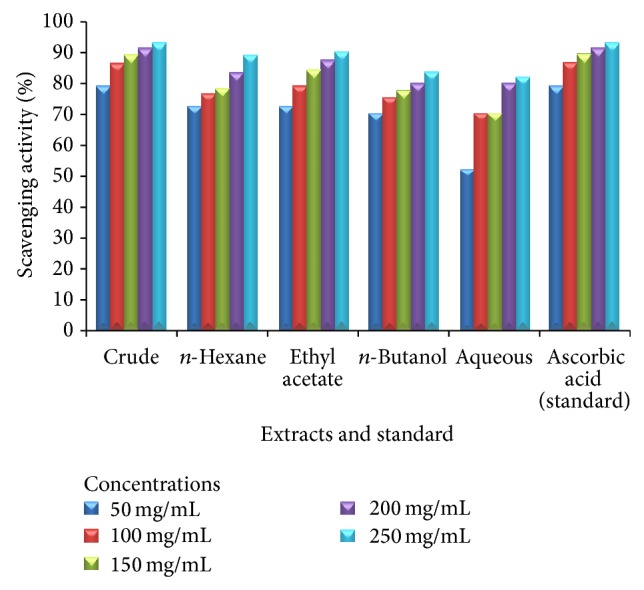
Antioxidant activity of various extracts of the leaves of* Heliotropium bacciferum* in comparison with the standard ascorbic acid.

**Figure 2:**
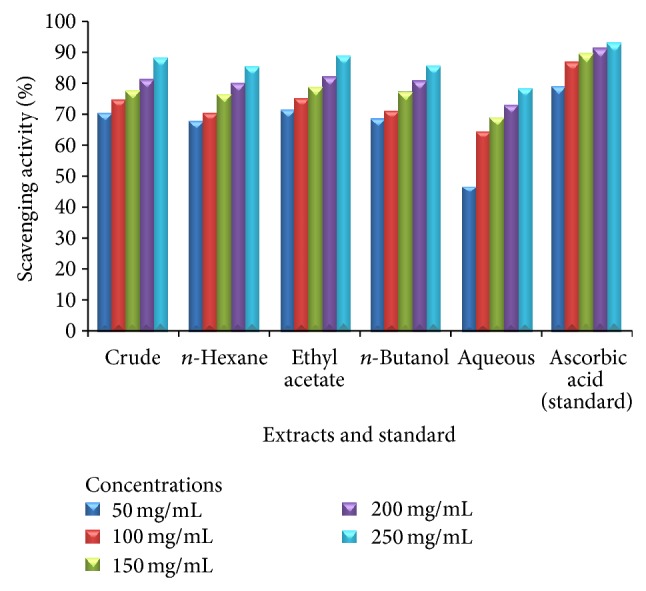
Antioxidant activity of various extracts of the stem of* Heliotropium bacciferum* in comparison with the standard ascorbic acid.

**Figure 3:**
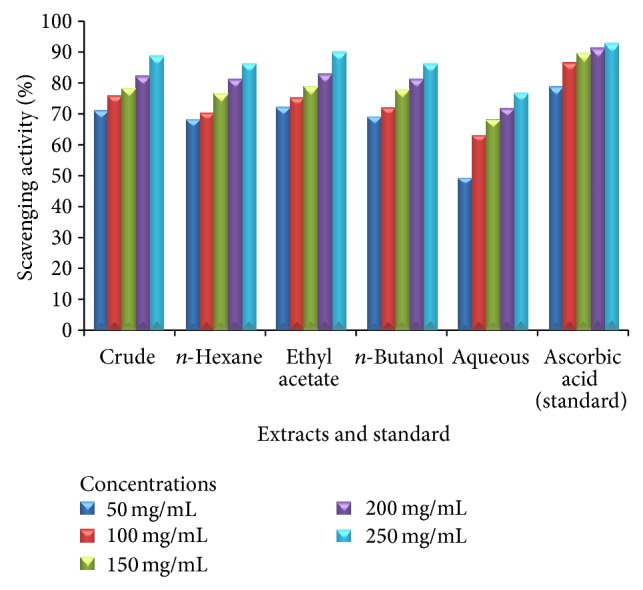
Antioxidant activity of various extracts of the roots of* Heliotropium bacciferum* in comparison with the standard ascorbic acid.

Tables 3, 4, and 5 demonstrate the antioxidant activities of the leaves, stem, and roots of plant* Heliotropium bacciferum*. Standard “ascorbic acid” exhibited significant DPPH radical scavenging activities. The plant leaves extracts revealed excellent DPPH radical scavenging activities ranging from 52.59 ± 0.84 to 90.74 ± 1.00 at concentrations of 50, 100, 150, 200, and 250 mg/mL, respectively (Figures 1, 2, and 3).

### 3.4. Fatty Acids Quantification of* Heliotropium bacciferum* by Gas Chromatography Mass Spectrometry (GC-MS)

**Figure 4:**
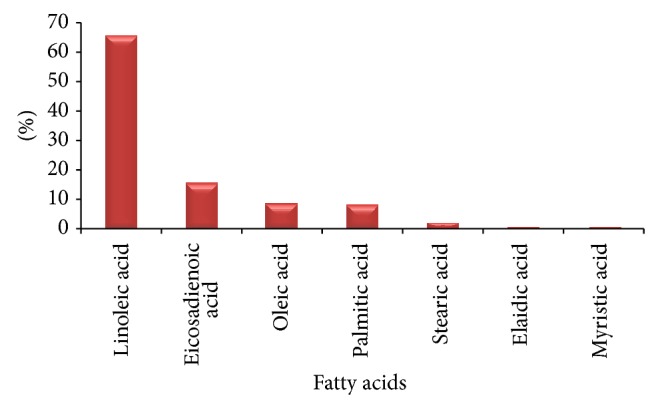
Quantitative analysis of fatty acids of* Heliotropium bacciferum* by GC-MS analysis.

**Figure 5:**
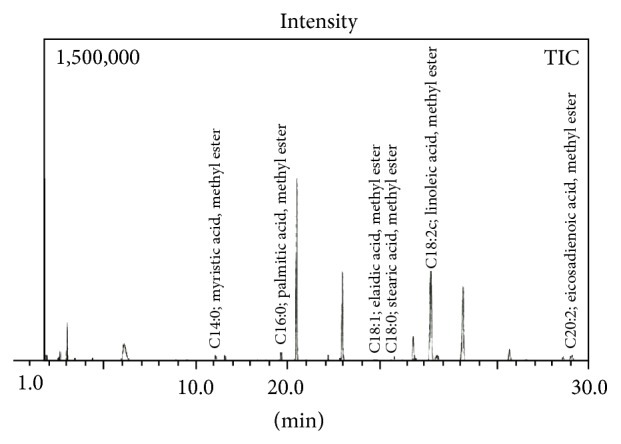
GC-MS chromatogram of the plant *n*-hexane extract with labeled signals detected by GC-MS detector (Analytes).

Table 6 viewing the names of fatty acids, area of relevant peaks, relative percentage compositions, times of the analysis, and retention time (R. time) was obtained from gas chromatography mass spectrometry (GC-MS) analysis. The percentage concentration and areas are the mean of the 3 measurements shown in Table 6.  Figure 5 shows the obtained GC-MS chromatogram of the *n*-hexane extract of the plant* Heliotropium bacciferum* with regularly labeled signals detected by GC-MS detector (Analytes). In the sample under investigation, the saturated and the unsaturated fatty acids were found (Figure 4).

### 3.5. FTIR (Fourier Transform Infrared) Spectroscopy

The infrared spectra of various extracts of the plant were recorded by IR Prestige-21 Fourier transform infrared spectroscopy (FTIR) and run under Infrared region of 400–4000 cm^−1^ range. From absorption spectra, the vibrational assignments, wave number (cm^−1^), and intensities of dominant peaks were recorded. The dominant IR peaks (see Figures  6–10 in the Supplementary Material available online at http://dx.doi.org/10.1155/2014/829076) of the plant signify the presence of different compounds such as aldehydes, alcohols, amides, ketones, ethers, and carboxylic acids. The more intense bands occurring at 2924 cm^−1^, 2998 cm^−1^, 2854 cm^−1^, 2853 cm^−1^, 1724 cm^−1^, 1489 cm^−1^, and 1230 cm^−1^ corresponding to the stretching or bending vibrations of O–H or N–H or C–H, C=O and C–Cl or C–S, respectively, signify the existence of amino acids, nitrates, alkenes, ethers, organic-halogen compounds, and carbohydrates.

## 4. Discussion

Plants containing steroids and flavonoid present in fruits and vegetables reduce the risk of atherosclerosis, which is build-up of fatty deposits in the artery walls [38]. Phenols and flavonoids in olive act as antioxidant, anticancer, antimicrobial, and antibacterial agents [39]. For compound identification, FTIR spectroscopy was used and run between the ranges of 400 and 4000 cm^−1^ under IR region. The peaks revealed that the plant has compounds such as amides, alcohol, aldehyde, ethers, ketone, and carboxylic acid [40].

Many herbs and plant species have been reported to possess DPPH radical scavenging activity. The plant* Heliotropium bacciferum* revealed significant DPPH radical scavenging activity. Other plants of genus* Heliotropium* also showed antioxidant activity. Plant aqueous fraction was primarily active. It has an EC_50_ value of 20.51 *μ*g/mL. Modak isolated three (3) flavonoids, 3-O-methylgalangin, 7-O-methyleriodictiol, and naringenin from the plant* Heliotropium taltalense*. The isolated flavonoids exhibited DPPH radical scavenging activity which recommends that* Heliotropium bacciferum* may possess flavonoids accountable for radical scavenging activity [41]. Phenolic compounds, for example, flavonoids, are of fastidious interest because of their antioxidant activity through oxygen radicals scavenging and peroxidation inhibition. Antioxidants that scavenge free radicals have a key role in inflammatory disorders, cancer, aging, and cardiovascular diseases [42]. Many antioxidant activities are due to the presence of coumarin lignans, flavonoids, flavones, anthocyanin, isocatechins, isoflavones, and catechins [43]. Heliotrine alkaloid demonstrated temporary hypotension perse in dogs and extensively condensed the nicotine induced vasopressor spasmogenic responses [44].

Drugs formulations on the basis of antioxidants are mostly used for the treatment and for the prevention of different diseases, such as Alzheimer's disease, stroke, cancer, diabetes, and atherosclerosis [45]. Some bacterial fatty acid profiles vary in composition according to external stimuli (temperature, pH, nitrogen source, salinity, etc.) [46]. In order to use specific fatty acid biomarkers to interpret environmental community structure, microorganisms should be examined for fatty acid patterns and their variation under different conditions. Taylor and Parkes showed that fatty acid profiles in some sulphate-reducing bacteria can be influenced by carbon source; however, in all cases major fatty acid biomarkers were identifiable [47]. Linoleic acid was found in highest percentage (65.70 ± 0.004%) in* Heliotropium bacciferum* followed by eicosadienoic acid (15.12 ± 0.002%), oleic acid (8.72 ± 0.007%), palmitic acid (8.14 ± 0.005%), stearic acid (1.74 ± 0.003%), elaidic acid (0.58 ± 0.002%), and myristic acid (0.20 ± 0.005%), respectively. In food nutrition evaluation, fatty acids have immense biological importance. In pharmacology and disease diagnosing, fatty acid also has key significance [48]. The unsaturated (monounsaturated or polyunsaturated) fatty acids are frequently used for declining heart disease risks, inflammation and increasing the immunity [14, 49].

## Supplementary Material

The supplementary materials: reveal the FTIR spectra of various plant extracts, which signify the presence of diverse compounds such as aldehydes, alcohols, amides, ketones etc.

## Figures and Tables

**Table 1:** Moisture, ash, and extractive values of the plant* Heliotropium bacciferum*.

Plant parts	Plant extracts	Extractive value (%) ± standard deviations	Moisture value (%)	Ash value of the whole plant (%)
Leaves	Methanol	32.64 ± 0.02	11.36 ± 0.04	8.67 ± 0.06
	*n-*Hexane	14.76 ± 0.03		
	Ethyl acetate	15.83 ± 0.02		
	*n-*Butanol	16.43 ± 0.04		
	Aqueous	23.79 ± 0.05		
Stem	Methanol	18.13 ± 0.05		
	*n-*Hexane	12.46 ± 0.01		
	Ethyl acetate	13.89 ± 0.03		
	*n-*Butanol	14.13 ± 0.10		
	Aqueous	20.10 ± 0.03		
Roots	Methanol	13.10 ± 0.08		
	*n-*Hexane	10.32 ± 0.03		
	Ethyl acetate	12.70 ± 0.06		
	*n-*Butanol	11.34 ± 0.12		
	Aqueous	17.16 ± 0.08		

**Table 2:** Phytochemical screening of various extracts of *Heliotropium bacciferum. *

Plant parts	Extracts	ALK	SAP	TAN	STE	TER	FLA	GLY	PHE
Leaves	Crude	+	+	+	+	+	+	+	+
	*n*-Hexane	+	−	+	+	−	+	−	+
	Ethyl acetate	+	+	+	−	+	+	+	+
	*n*-Butanol	+	+	−	−	+	+	−	+
	Aqueous	+	+	−	−	+	−	+	−

Stem	Crude	+	+	+	+	+	+	+	+
	*n*-Hexane	+	−	+	+	−	−	−	+
	Ethyl acetate	+	+	+	−	+	−	+	+
	*n*-Butanol	+	+	−	−	−	+	−	+
	Aqueous	+	+	−	−	+	+	−	+

Roots	Crude	+	+	+	+	+	+	+	+
	*n*-Hexane	+	−	−	+	−	+	−	−
	Ethyl acetate	+	+	+	−	+	+	−	+
	*n*-Butanol	+	+	−	−	−	+	−	+
	Aqueous	+	+	−	−	+	−	+	−

(+): present; (−): absent; ALK: alkaloids, SAP: saponins, TAN: tannin, STE: steroids, TER: terpenoids, FLA: flavonoids, GLY: glycosides, and PHE: phenols.

**Table 3:** *In vitro* antioxidant activities of all the extracts of *Heliotropium bacciferum* (leaves).

Extracts	Quantity in milligram (mg/mL), mean value ± standard deviation				
	50	100	150	200	250
Ascorbic acid (standard)	79.12 ± 0.81	86.79 ± 0.33	89.84 ± 0.72	91.51 ± 0.41	93.22 ± 0.58
Crude	72.57 ± 0.94	76.97 ± 0.89	78.89 ± 0.59	83.63 ± 0.57	90.18 ± 0.90
*n-*Hexane	67.83 ± 1.02	73.47 ± 0.94	81.48 ± 0.73	87.13 ± 0.87	89.19 ± 0.53
Ethyl acetate	72.57 ± 0.71	79.23 ± 0.55	84.90 ± 0.76	87.58 ± 0.99	90.74 ± 1.00
*n-*Butanol	70.65 ± 0.34	75.95 ± 0.48	78.21 ± 0.98	80.47 ± 0.70	84.31 ± 0.92
Aqueous	52.59 ± 0.84	69.97 ± 0.76	70.76 ± 0.42	80.02 ± 0.32	82.73 ± 0.47

**Table 4:** *In vitro* antioxidant activities of all the extracts of *Heliotropium bacciferum* (stem).

Extracts	Quantity in milligram (mg/mL), mean value ± standard deviation				
	50	100	150	200	250
Ascorbic acid (standard)	79.12 ± 0.81	86.79 ± 1.33	89.84 ± 0.72	91.51 ± 0.41	93.22 ± 0.58
Crude	70.34 ± 0.82	74.78 ± 0.73	77.72 ± 1.07	81.57 ± 0.87	88.13 ± 0.49
*n-*Hexane	67.83 ± 1.02	70.39 ± 0.71	76.32 ± 0.63	80.17 ± 1.01	85.29 ± 0.65
Ethyl acetate	71.63 ± 1.51	74.98 ± 0.95	78.90 ± 1.02	82.34 ± 0.88	89.42 ± 1.10
*n-*Butanol	68.53 ± 0.90	71.31 ± 1.38	77.01 ± 0.98	80.98 ± 0.60	85.79 ± 1.21
Aqueous	50.19 ± 0.92	64.37 ± 0.62	69.06 ± 1.42	73.02 ± 0.12	78.43 ± 0.70

**Table 5:** *In vitro* antioxidant activities of all the extracts of *Heliotropium bacciferum* (roots).

Extracts	Quantity in milligram (mg/mL), mean value ± standard deviation				
	50	100	150	200	250
Ascorbic acid (standard)	79.12 ± 0.81	86.79 ± 1.33	89.84 ± 0.72	91.51 ± 0.41	93.22 ± 0.58
Crude	71.14 ± 0.29	75.88 ± 1.03	78.82 ± 1.01	82.17 ± 0.63	88.89 ± 0.39
*n-*Hexane	68.13 ± 1.12	70.19 ± 1.1	76.892 ± 0.13	81.17 ± 1.01	86.19 ± 0.15
Ethyl acetate	72.13 ± 1.03	75.38 ± 0.81	79.10 ± 0.12	83.24 ± 0.38	90.01 ± 1.02
*n-*Butanol	69.13 ± 1.00	72.11 ± 1.18	78.01 ± 0.12	81.28 ± 0.49	86.21 ± 1.01
Aqueous	49.19 ± 0.52	63.38 ± 1.62	68.16 ± 1.32	72.13 ± 0.42	77.03 ± 1.30

**Table 6:** Quantitative results of fatty acids of *Heliotropium bacciferum *by GC-MS analysis.

S. number	Name	R. time^*α*^	Area^*^	Percentage^*^	Std. Dev.^*β*^
1	C18:2c; linoleic acid	21.361	95520	65.70	0.004
2	C20:2; eicosadienoic acid	21.739	23034	15.12	0.002
3	C18:1c; oleic acid	20.155	12574	8.72	0.007
4	C16:0; palmitic acid	14.618	51990	8.14	0.005
5	C18:0; stearic acid	19.628	9500	1.74	0.003
6	C18:1; elaidic acid	20.392	638	0.58	0.002
7	C14:0; myristic acid	10.955	1242	0.20	0.005

^*α*^Retention time, ^*^average of three (3) measurements, and ^*β*^standard deviation of the three measurements.
