# Supplementary material for: Phytochemical Analysis, Antioxidant Activity, Fatty Acids Composition, and Functional Group Analysis of Heliotropium bacciferum
Source: ScientificWorldJournal. 2014 Nov 12;2014:829076. doi: 10.1155/2014/829076 (PMC4247971; doi:10.1155/2014/829076)
Supplement: Supplementary file 1 — The supplementary materials: reveal the FTIR spectra of various plant extracts, which signify the presence of diverse compounds such as aldehydes, alcohols, amides, ketones etc. [file 829076.f1.docx]

**Supplementary Material**

FIGURE 6. FTIR spectroscopy of Crude extract of *Heliotropium bacciferum*

FIGURE 7. FTIR spectroscopy of *n-*hexane extract of *Heliotropium bacciferum*

FIGURE 8. FTIR spectroscopy of Ethyl acetate extract of *Heliotropium bacciferum*

FIGURE9: FTIR spectroscopy of *n-*butanol extract of *Heliotropium bacciferum*

**
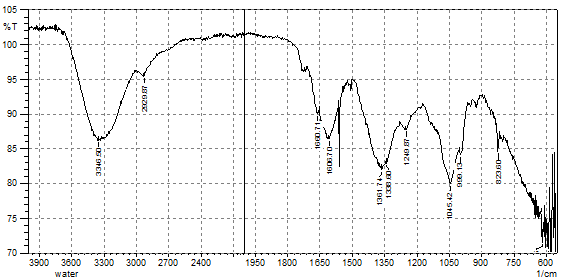
**

FIGURE 10**.** FTIR spectroscopy of aqueous extract of *Heliotropium bacciferum*
